# A DIGITAL INTERVENTION TO PROMOTE EFFECTIVE COPING DURING CRISIS AND ROUTINE AMONG OLDER ADULTS

**DOI:** 10.1093/geroni/igad104.0742

**Published:** 2023-12-21

**Authors:** Stav Shapira, Daphna Yeshua-Katz, Avital Avigezer, Su-I Hou, Orly Sarid

**Affiliations:** Ben-Gurion University of the Negev, Beer Sheva, HaDarom, Israel; Ben-Gurion University of the Negev, Beer Sheva, HaDarom, Israel; Ben-Gurion University of the Negev, Beer Sheva, HaDarom, Israel; School of Global Health Management & Informatics, University of Central Florida, Orlando, FL, USA, Oviedo, Florida, United States; Ben-Gurion University of the Negev, Beer Sheva, HaDarom, Israel

## Abstract

**Introduction:**

We developed and evaluated a short-term group intervention aimed at promoting effective coping and alleviating adverse mental health impacts during the first wave of COVID-19 in Israel. The intervention consists of seven sessions, each including: a guided group discussion, and learning and practicing cognitive-behavioral and mindfulness techniques.

**Methods:**

In this pilot randomized controlled trial, we recruited eighty-two community-dwelling older adults who were assigned to either an intervention group (n=64) or a wait-list control group (n=18). Participants completed questionnaires assessing depression, loneliness, subjective health, and social support. We collected measurements before (T0), immediately after participation (T1), 4 weeks after participation (T2), and 1.5 years after participation in the intervention (T3).

**Results:**

Our findings revealed statistically and clinically significant reductions in depression in the intervention group, with results maintained at one-month and 1.5-year follow-ups. While loneliness levels also significantly decreased post-intervention, this benefit was not maintained at follow-up measurements. Social support and subjective health did not change significantly. The wait-list control group did not show any overall changes. Participants reported frequently using cognitive reconstruction and positive self-talk techniques at the 1.5-year follow-up. The current degree of technique use/practice significantly predicted depression level at T3.

**Conclusions:**

Our intervention presents a simple and easy-to-implement tool with broad relevance beyond the COVID-19 pandemic, as the skills acquired also positively influence mental health outcomes during routine life. This relatively simple model can be effectively utilized by communities globally to help connect lonely and isolated older inhabitants and promote effective coping with various stressful situations.

